# Shadow Education and Its Academic Effects in Bangladesh: A Vygotskian Perspective

**DOI:** 10.3389/fpsyg.2022.922743

**Published:** 2022-07-12

**Authors:** Md. Bayezid Alam, Zhiyong Zhu

**Affiliations:** ^1^Department of Political Science, Murarichand College, Sylhet, Bangladesh; ^2^Faculty of Education, Beijing Normal University, Beijing, China

**Keywords:** scaffolding, private tutoring, shadow education, Vygotskian perspective, Bangladesh

## Abstract

Private tutoring is a newly emerging field of research, which remains largely under-theorized. Adopting the Vygotskian philosophy of learning as an analytical lens, this qualitative study conceptualized the academic effects of private tutoring in a Bangladeshi higher secondary educational context. The primary data were gathered from 18 semi-structured interviews with tutored students, parents, and teachers. The data from secondary sources were also collected to supplement the primary data. A thematic procedure was used to analyze the data. The analysis demonstrated two important findings. First, students internalized knowledge and skills through scaffolding by their private tutors, which eliminated their learning deficiencies and boosted their academic credentials. Second, private tutoring induced long-term deficiencies as it provided rote learning. It dulled students’ critical thinking and made them dependent on others. The paper carried significant theoretical implications by producing a unique insight into students’ learning in the shadows from a Vygotskian perspective.

## Introduction

Private tutoring, widely known as shadow education, has emerged as a parallel to mainstream schooling (Baker et al., 2001, p. 3; Dang and Rogers, 2008, p. 161; Mahmud, 2018, p. 343). It refers to fee-based educational activities that occur outside of mainstream schooling but follow the same curriculum (Stevenson and Baker, 1992, p. 1639; Kumar and Chowdhury, 2021, p. 245). Students and their parents generally seek such tutoring services either for remedial or enrichment purposes (Zhang et al., 2021, p. 240; Mahmud and Kenayathulla, 2018, p. 704; Hamid et al., 2018, p. 882). Private tutoring is more prevalent in societies where the education system is highly competitive and high-stake testing plays a crucial role (Atalmis et al., 2016, p. 1136; Zhang et al., 2021, p. 239; Ömeroğulları et al., 2020). More recently, it has grown rapidly across the globe, but there is considerable transnational variation in its application.

Private tutoring is increasingly prevalent in South Asia because of the competitive nature of evaluation (Borkowski et al., 2021, p. 99) and aspiration for upward social mobility (Joshi, 2019, p. 16). Bangladesh has a long history of private tutoring culture (Alam and Zhu, 2022, p. 20), and has recently been practiced on an alarming scale. For instance, 92 percent of Bangladeshi students were reported to have received private tutoring in 2017, which was highest among the Asia-Pacific countries (UNESCO, 2018, p. 72). The country’s education system is highly competitive (Akter, 2021, p. 35) and formal schooling has deep-rooted deficiencies that contributed to the expansion of private tutoring (Khatun and Saadat, 2018, p. 3). Thus, many students rely entirely or partially on supplementary tutoring provided by regular teachers or even non-teachers (UNESCO, 2018, p. 72).

Bangladeshi “teachers are significantly under-paid” (Asadullah, 2006, p. 1044), so many of them supplement their income by tutoring students outside of school hours (Austin et al., 2008, p. 23; Hera, 2020, p. 7; Nayeem, 2017, pp. 6–7). Although private tutoring compensates teachers financially, it raises certain ethical concerns (Anwaruddin and Pervin, 2015, p. 31). Instead of teaching professionally in regular classes, the teachers force their students to take private lessons from them (Choe et al., 2013, p. 240) and often provide preferential treatment to the tutored students (Ahmed, 2017). Students must spend extra to receive the best services and achieve the best examination outcomes (Hera, 2020, p. 7). Private tutoring makes education expensive for low-income families (Choe et al., 2013, p. 240). The policy, however, prohibits teachers from privately tutoring their own institution’s students while allowing them to instruct up to 10 students belonging to other institutions a day with prior consent from both the student and the teacher’s institutions (Bray and Kwo, 2014, p. 45; Joshi, 2019, p. 13; Borkowski et al., 2021, p. 195). The restrictions only existed on paper, in fact. Mainstream teachers are still providing supplemental tutoring in full force (Ahmed, 2017).

Given the growing prevalence of private tutoring, it has been widely studied in different settings around the globe, yielding varied results in terms of its effectiveness (Dang and Rogers, 2008, pp. 172–174). However, private tutoring is still a “relatively under-researched area” in Bangladesh (Hamid et al., 2009, p. 303). Specifically, there is a lack of rigorous studies that empirically investigate the effects of private tutoring on Bangladeshi students’ educational outcomes. Furthermore, as a newly emerging field of research, private tutoring remains largely under-theorized. Adopting the Vygotskian philosophy of learning as an analytical lens, this study conceptualized the academic effects of private tutoring among Bangladeshi higher secondary students. The study addressed the following two research questions: (1) Does private tutoring lead to academic benefits for higher secondary students in Bangladesh? (2) Does private tutoring initiate any long-term deficiencies for higher secondary students’ learning in Bangladesh?

## Literature Review

### Shadow Education and Its Academic Effects

Private tutoring is usually seen as one of the contributing factors to a positive learning outcome (Brehm and Silova, 2014, p. 109; Song et al., 2013, p. 128). Students are expected to learn more and perform better in the examinations if they devote more time to studying the subjects (Berberoğlu and Tansel, 2014, p. 687). Mischo and Haag (2002, p. 263) in Germany found that private tutoring significantly affects students’ scores on teacher-designed tests. Other German studies (e.g., Guill and Bos, 2014, pp. 54–55), on the other hand, did not find a significant effect of private tutoring on students’ academic achievement. Berberoğlu and Tansel (2014, p. 697) in Turkey reported substantial positive effects in both language and mathematics.

Stevenson and Baker (1992, p. 1639) demonstrated that private tutoring enhanced the probability of Japanese high school students entering an elite university. Byun (2014, pp. 54-55) reported that attending cram school had a significant positive effect on academic achievement among South Korean students, whereas other forms (e.g., individual tutoring, online tutoring) did not. Ha and Park (2017, p. 65) showed that private tutoring contributed to improving the academic achievement of secondary school students. In contrast, Lee et al. (2004, p. 25) indicated that tutoring in Korean, English, and mathematics had no significant effects on students’ academic achievements. Choi et al. (2012) reported that the effect of private tutoring was “positive for mathematics, positive but decreasing for reading, and non-significant for science (p. 299).”

Zhang (2013) investigated the associations between private tutoring and students’ scores on the National College Entrance Exam (NCEE) in Jinan, China. She demonstrated that although private tutoring did not significantly affect students’ NCEE outcomes as a whole, it had considerable effects on particular subgroups (e.g., English test scores). Liu (2012, p. 46) in Taiwan outlined the positive effects of cram schooling on students’ analytical ability and math performance. The positive effects, however, declined when cramming hours were extended. Kuan (2018, p. 391) observed that cramming programs have a positive effect on 9^th^ grade students’ academic achievement. Cheo and Quah (2005, p. 280) in Singapore found negative effects, whereas Loyalka and Zakharov (2016, p. 29) in Russia reported mixed findings, as private tutoring had significant effects on high-achieving students and insignificant effects on low-achieving students.

Banerjee et al. (2007, p. 19) in India and Herath (2022) in Sri Lanka found a strong positive contribution of private tutoring on students’ academic achievement. Azmat et al. (2021, p. 277) in Pakistan claimed that private tutors supplied notes/suggestions and focused on drill learning, which helped the students in gaining higher examination scores rather than focusing on critical thinking skills. A very few Bangladeshi studies have looked into whether private tutoring increases students’ academic ability. Shihab and Sultana (2017, p. 9), for example, noted positive effects of private tutoring on English among secondary students. Hamid et al. (2009, p. 300) demonstrated positive but weak associations between students’ tutoring participation and their English achievement. Mahmud (2019) indicated that private tutoring had mixed impacts on students’ learning. It reduced students’ learning difficulties and increased their learning attainment. It also had some drawbacks due to the examination-centered aim.

Prior studies arrived at different conclusions regarding the academic effects of private tutoring (Ömeroğulları et al., 2020, p. 1). Some studies relied on data from self-reported student achievement, while others relied on data from cognitive tests (Zhang and Liu, 2016, p. 36), and there were methodological and contextual differences (Berberoğlu and Tansel, 2014, p. 687). Thus, the existing studies produced inconclusive, contradictory, and even confusing findings (Byun, 2014, p. 40; Bray, 2014, p. 381). Moreover, private tutoring is a newly emerging field of research, which remains largely under-theorized. The current study therefore adds to the body of knowledge by reporting the academic effects of private tutoring among Bangladeshi higher secondary students, using Vygotsky’s philosophy of learning.

### Scaffolding for Learning: Theoretical Lens

**Figure 1:**
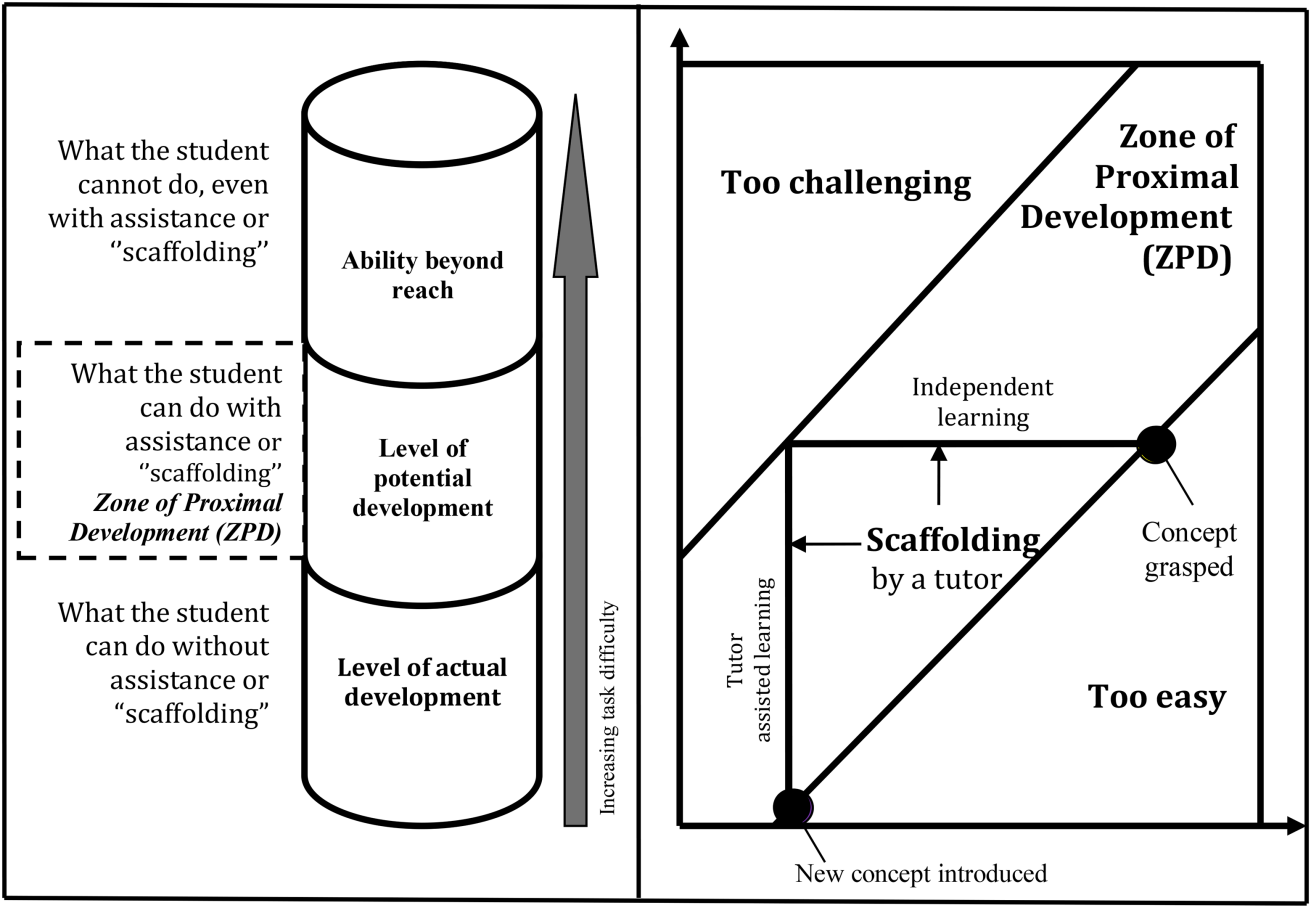
The zone of proximal development and scaffolding. Source: Modified from Vadnais (2015) and Grahame (2020).

Vygotsky’s view of learning has already gained paramount attention in the field of education. He stipulated that learning occurs in the “zone of proximal development” (ZPD; Mady, 2019, p. 17; Verenikina, 2010, p. 17). The students who are in the ZPD cannot do the task unless they are assisted by a “more knowledgeable other” (MKO). Vygotsky defined ZPD as the imaginary distance between students’ actual and potential level of development (Vygotsky, 1978). Actual development refers to students’ current capability to carry out a task independently (Palincsar, 1998, pp. 352–353). By contrast, what students can achieve with scaffolding (temporary assistance) is hypothesized as the potential level of development (Lui, 2012, p. 3). The MKO provides the scaffolds so that students can accomplish the task within the ZPD (Fani and Ghaemi, 2011, p. 1550; Daw, 2011, p. 11). Vygotsky’s notions of ZPD, scaffolding, and MKO are shown in Figure 1.

The scaffolds include demonstrating, explaining, summarizing, questioning, answering, and suggesting (Haider and Yasmin, 2015, p. 170; Arshad and Chen, 2009, p. 328). The MKO gradually withdraws scaffolding until the students can perform the task independently. Some critics argue that Vygotsky’s “ZPD presents a restricted view of learning processes” (Lambert and Clyde, 2000, p. 29) and that his theory disregards the individuals’ role in learning (Lui and Matthews, 2005, p. 391). Furthermore, Vygotsky viewed learning as a result of active construction, but sometimes “learning can occur passively or osmotically” (Zhou and Brown, 2017, p. 36).

Vygotsky’s view can be applied to gain insight into students’ learning in the shadows. Here, MKO refers to a private tutor who has a better understanding of the topic being taught (Esther et al., 2017, p. 1325). Private tutors can mediate students’ competence within the ZPD. Students can internalize new knowledge and can enhance their skills through scaffolding by the private tutors that they cannot do individually. The scaffolding provided by the private tutors is temporary. It is gradually withdrawn since the students’ abilities are enhanced.

### Bangladeshi Higher Secondary Educational Context

Higher secondary education (Grades 11 and 12) in Bangladesh comprises two-years of formal schooling for the age group of 16 to 17 years that links between secondary and tertiary education. After successfully completing this stage, students face a high-stakes university entrance examination. The higher secondary courses are diversified into different specializations or tracks. Both public and private categories of institutions offer education at this level. Although higher secondary education includes three major streams (e.g., general, *madrasah* [religious], and Technical-Vocational Education and Training [TVET]), the current study only focuses on the general stream.

Bangladeshi education is highly competitive, stress-fueled, and examination-driven. Two types of examinations (e.g., internal and public) are held at the higher secondary level. The colleges primarily conducted annual and test examinations at the end of Grade 11 and Grade 12, respectively, (Hoque et al., 2007). The students participate in an annual examination to promote from Grade 11 to Grade 12, while the test examination is a requirement for sitting in the public examination. The public examination, known as the Higher Secondary Certificate (HSC), is administered by the Board of Intermediate and Secondary Education (BISE) at the end of each cycle (Hoque, 2011). The high-stakes HSC examination forced students to concentrate on private tutoring for the development of examination skills.

Bangladesh Bureau of Educational Information and Statistics’s (BANBEIS, 2019) data showed that both the pass rate and the number of GPA-5 (grade point average, the highest result) achievers in the HSC examination increased noticeably over the years. Between 2004 and 2018, the total number of examinees climbed by 2.22 times—from 4,83,481 to 10,72,028. The pass rates rose dramatically—from 47.73 percent in 2004 to 74.85 percent in 2008. Pass rates fluctuated between 65 to 77 percent during the period 2009–2018. Several factors were responsible for these fluctuations, such as changes to textbooks, examination formats, and assessment mechanisms. The number of students getting GPA-5 was only 3,036 in 2004, whereas this number stood at 25,562 in 2018. The number of students passing the examination had only increased by 3 times, whereas the number of students getting GPA-5 jumped by 8.42 times between 2004 and 2018.

## Methodology

The study adopted a qualitative approach to gain an in-depth and holistic picture of shadow learning in Bangladesh (Merriam, 1998). It helped the researcher to explore the phenomenon in a “natural setting” (Denzin and Lincoln, 1994), where respondents had actually lived and experienced (Creswell, 2007), and to interpret the subjective meanings from the respondents’ perspectives (Muijs, 2004). Two urban public institutions (College A and College B) were purposefully selected as research sites because of their uniqueness and easy access. Apart from the sites’ easy access, three other specific uniquenesses were considered to decide their selection: Firstly, both of the colleges were information-rich. Secondly, no studies have been conducted in these institutions, although their students consume a high rate of private tutoring. Thirdly, both of the colleges were considered to have a high level of “education zeal” in the Sylhet division, a north-eastern part of Bangladesh. Due to limited resources and time constraints, this study concentrated solely on higher secondary education, a critical period of students’ academic life in Bangladesh.

### Participants

A total of 18 participants were recruited, comprising 6 tutored students, 6 teacher-tutors, and 6 parents. The participants were chosen based on their potential to generate data relevant to the purpose of the study. The researcher first purposefully selected six teacher-tutors who were actively involved in private tutoring. Selected teachers later helped to find the students who received tutoring from them. Patton’s (2015) “maximum variation sampling” technique was used in the selection of six tutored students and their concerned parents since it maximized the heterogeneity of samples and provided relatability to a broad audience. Student participants had the maximum variation in terms of their gender, grade, and academic ability (high-achiever, mid-achiever, and low-achiever). Parent participants were recruited considering their gender, educational level, and occupation. Pseudonyms were used to maintain the anonymity of the participants. Participant selection and data collection took place between November 2018 and August 2019. Key information about the participants is illustrated in Table 1.

**Table 1:** Profile of the participants.

Pseudonym	Group	Study site	Gender	Education level	Interview setting	Ability/profession/experience
S1	Student	College A	Male	Grade 11	In restaurant	High-achiever
S2	Student	College A	Male	Grade 12	In cafe	Mid-achiever
S3	Student	College A	Male	Grade 11	At home	Low-achiever
S4	Student	College B	Female	Grade 12	In tutor’s home	Mid-achiever
S5	Student	College B	Female	Grade 11	In campus	Low-achiever
S6	Student	College B	Male	Grade 12	In tutor’s home	High-achiever
P1	Parent	College A	Male	Master	At home	Banker
P2	Parent	College A	Female	Bachelor	At home	Business
P3	Parent	College A	Female	HSC	At home	Homemaker
P4	Parent	College B	Male	Bachelor	In office	Lawyer
P5	Parent	College B	Male	Bachelor	In library	Business
P6	Parent	College B	Male	Bachelor	In restaurant	Business
T1	Teacher	College A	Male	Master	At home	15 years
T2	Teacher	College A	Male	Master	At home	7 years
T3	Teacher	College A	Male	Master	At home	8 years
T4	Teacher	College B	Male	Master	At home	15 years
T5	Teacher	College B	Male	Master	In restaurant	10 years
T6	Teacher	College B	Male	Master	At home	10 years

### Data Collection

The primary data were collected through 18 face-to-face semi-structured interviews. The researcher first communicated with potential participants over the phone and clearly explained the research intentions. Each participant was then interviewed separately according to their convenient time and place. Prior to starting each interview, written consent was obtained from the participants. They were informed about their right to withdraw themselves from the study at any time and were assured that their responses would be treated as confidential. The researcher tried to create a relaxing and friendly atmosphere so that the participants could express their in-depth perceptions without any constraints. The interviews lasted between forty minutes to one hour and were audio recorded prior to the permission of the participants. Data from secondary sources (e.g., scholarly articles, reports, and documents) were collected to supplement primary data. Secondary data could not only enrich the data set but also triangulate the data and add to the trustworthiness of this qualitative study.

### Data Analysis

**Figure 2:**
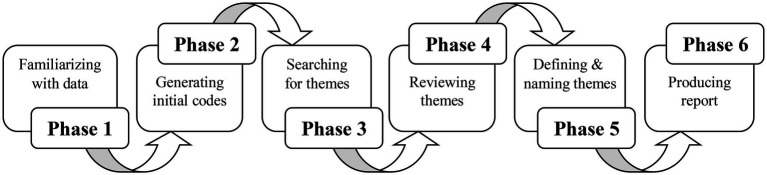
Six-step thematic analysis procedure. Source: Braun and Clarke (2006).

Braun and Clarke’s (2006) thematic procedure was followed to analyze the data (see Figure 2). This method provided a rich account of the data and generated unanticipated insights. The researcher first became familiar with the data set through transcribing the data, reading the data repeatedly, and noting down initial ideas in the margins. Once familiar with the data, the initial codes were generated that represented important features of the data. When all the data had been coded, the researcher looked for similarities among the codes and chunked them into potential themes. Then the themes were reviewed, modified, or discarded to make them fit the data better. During the next phase, the themes were defined and the comprehensive names were given. Once the themes were fully established, the researcher began to write-up the report.

## Findings

### Eliminating Learning Deficiencies

Private tutoring provided academic assistance to the students in terms of remediation. Actually, students could not get what they needed from the classroom due to inadequate instruction (Quadir, 2019, p. 82). Private tutors address their learning deficiencies in classroom studies. All student interviewees (S1, S2, S3, S4, S5, S6) reflected that private tutoring helped them to meet their expected needs, ask their tutors any questions, measure their progress clearly, and complete their syllabus in due time. As a whole, they eliminated their academic weaknesses through scaffolding by the private tutors, as one student said:

We could not understand what our teachers delivered in their lectures because there were a lot of students in the classroom. This created a learning gap that could be filled through private tutoring (S4).

Another student (S3) expressed his dissatisfaction with the pedagogies followed by mainstream teachers. As different teachers taught different topics, he could not understand the lessons clearly. He filled such kinds of deficit through scaffolding by the private tutors. All teacher interviewees (T1, T2, T3, T4, T5, T6) perceived that they helped students fill their learning gaps. One teacher (T4) stated that he could satisfy his students in private tutoring, but not in the classroom. As a private tutor, he repeatedly explained the topics to make the students understand. Another teacher indicated:

A teacher cannot teach a student repeatedly in a class due to insufficient time. At the same time, teaching is a gradual process. Sometimes, a student might not come to the class… Some students may always be absent due to illness or family problems. They resort to private tutoring to meet these deficiencies (T6).

Similarly, all parent interviewees (P1, P2, P3, P4, P5, P6) opined that private tutors helped their children to strengthen their learning capabilities. As there were fewer students in private tutoring, they got incentive care from the tutors. In fact, private tutoring kept students on track with their studies. Without private tutoring, they would be unable to continue their studies in the current reality. Secondary data showed that private tutors assisted their students in filling in the gaps or solving hard problems related to what they had not learned in formal school (Manzoor, 2013, p. 54). Furthermore, learning assistance is required for the weaker students, which can be successfully offered by private tutors (Hossain, 2018, p. 50). Thus, Nath (2008, p. 55) claimed that students who received private tutoring learned more than those who did not.

### Improving Examination Scores

Examinations are an important part of Bangladesh’s education system (Amin and Greenwood, 2018, p. 13), with students spending more than 7 hours per week, both in and out of the classroom, preparing for examinations (UNESCO, 2018, p. 47). Indeed, private tutoring was viewed as a nice place for examination preparation. All student interviewees (S1, S2, S3, S4, S5, S6) claimed that they improved their examination scores because of private tutoring (see Table 2). They received many shortcuts techniques, hand-notes, and suggestions from private tutors, which helped them to cut a good figure in the examination. Those who did not receive tutoring would not get these benefits. One student (S3) claimed that science teachers prepared some fixed notes for a practical examination. Students passed such an examination by easily memorizing these notes. Another student shared her views as:

**Table 2:** The effects of private tutoring on examination scores.

Students	Responses
S1	“Although there are some negative effects of private tutoring, our results are improving.”
S2	“When we go to the tutors’ house and finish the syllabus with satisfaction, we do not have any shortage. They solve our problems taking much time. Hence, our results are getting better.”
S3	“Private tutoring certainly helps to get better results. Because, the parts where the questions come from in the examination are taught with importance.”
S4	“Sir [private tutor] used to provide suggestions, and many questions were common from those suggestions in internal examinations. As a result, I got good marks.”
S5	“As a result of understanding differently from Sir (private tutor), we are able to answer well in the examination script.”
S6	“Private tutoring is playing a positive role in getting a better GPA.”

There is a relationship between results and private tutoring. I would not have understood my lesson better if I had not studied with any private teacher. I would not have gotten any suggestions. So, my result would be poor (S4).

All parent interviewees (P1, P2, P3, P4, P5, P6) perceived private tutoring as inevitable for their children’s examination scores. Due to intensive care from a skilled tutor, their children could strengthen their preparations for examinations. One parent (P1) mentioned that many teachers stopped tutoring practices when the government enacted a regulation in 2012. Students suddenly became directionless and could not receive tutoring for a few months. Therefore, the results for the HSC examination deteriorated in that year. Another parent stated:

In private tutoring, teachers teach systematically, take tests frequently, and give feedback. If someone studies a lot the whole year, knows a lot but cannot present them properly in the examination, then will it be possible to get a better result? Students need to be technical for better results. Private tutors… give them numerous tips. Their aim is to get students’ better results; otherwise they will not get students the next year (P3).

All teacher interviewees (T1, T2, T3, T4, T5, T6) noted that private tutoring is more examination-centric, which polished students for better scores. If they stopped tutoring, the students’ results would be a downfall. To make the students suitable for the examination, they gave self-made notes, suggestions, and various shortcut techniques. Students were making good figures in the examination with very little studying. Some teachers (T4, T5, T6) claimed that they could take extra classes before examinations, and make students practice the problems more. They noticed the differences in results for those who took tutoring and those who did not. The students who receive tutoring really get better results.

Higher secondary students are concerned with meeting their expectations for examination results (UNESCO, 2018, p. 66). To succeed in the HSC examinations, they received scaffolding instruction from the private tutors. Naka (2013, p. 288) indicated that private tutoring is often crucial for Bangladeshi students to achieve good scores on the examination. Rahman et al. (2019, p. 24) pointed out that tutored students increased their mathematics, Bengali, and English scores by 3.2, 2.7, and 2.3 percentage points, respectively, when compared to non-tutored students.

### Affecting Next-Level Education

Higher education is extremely competitive in Bangladesh. Only a small percentage of total applicants are chosen for admission to public universities. In 2016–17, for example, 302,489 admission seekers applied for 6,800 seats at the University of Dhaka, resulting in 45 applicants per seat (UNESCO, 2018, p. 82). Most student interviewees (S1, S2, S3, S5, S6) perceived that private tutoring affected their next-level education. Tutoring played a role in having a better score in the HSC examination that would give them a qualification to sit for the university entrance test. Moreover, private tutors provided some guidelines, shortcut techniques, and motivations that were certainly helpful to survive in the competitive examinations. As one student commented:

By receiving tutoring, we become qualified to sit for competitive examinations… The tutors teach us all the basics and give tips for university admission tests as well… I have bought some extra books in accordance with my private tutor’s direction and have benefited greatly. Private tutors motivate students a lot. I have been inspired by their motivation and have pledged to survive. I will… I have built great confidence due to private tutoring (S6).

By contrast, one student (S4) argued that receiving tutoring at a higher secondary level would not affect her chances of next-level education. It was possible to pass the HSC examination by studying the notes and suggestions gotten from private tutoring. But the pattern of university entrance tests was completely different from the higher secondary curriculum. Four parent interviewees (P1, P3, P4, P6) believed that private tutoring would contribute to their children’s university entrance tests and work-life competition. One parent noted:

Private tutoring would have a positive impact on my son’s future life. My son would cut a good figure in the HSC examination and would get the chance of admission in a prestigious university, as students’ previous results are considered during the university entrance test (P1).

By contrast, two parent interviewees (P2, P5) perceived that tutoring would not affect their children’s future. Rather, they argued that getting the chance to go to university has become an uncertain matter. They claimed that many students of their generation achieved good results without private tutoring and later became renowned bureaucrats, university teachers, and scientists. Most teacher interviewees (T2, T3, T5, T6) reflected that tutoring generated learning habits and helped in organizing knowledge that increased the chances of getting better education and jobs. For example, one teacher claimed that many of his students were studying at different universities, medical and engineering colleges. His motivational comments in private tutoring encouraged his students greatly:

Students’ foundation for higher education and work life is built at the higher secondary level. Due to a lack of time, I cannot motivate students that much in the classroom. But I have been spending quite a lot of time with students in my private tutoring… I have lots of opportunities here to motivate them. And students develop the mentality to receive that motivation (T6).

By contrast, one teacher (T4) indicated that many poor students are admitted to university and later guaranteed jobs without receiving any tutoring. Another teacher (T1) claimed that private tutoring was creating a weak foundation for students. They were making a better score in HSC examination by learning some selected topics in the tutors’ house, but they often faced difficulties to pass the university entrance test and job market competitions. Or they arrived at a ready platform using their patriarchal or social position.

### Downgrading Critical Thinking Skills

Critical thinking is extremely important to successful academic performance (Akhter, 2019, p. 130). Students with excellent critical thinking skills will be better equipped for a future full of challenges and opportunities. Private tutoring neglects the essence of students’ actual learning (Kawedhar et al., 2020, pp. 225–226) as it promotes rote learning. Private tutors did not teach in detail, they only focused on important topics for examinations which in fact initiated long-term deficiencies. All student interviewees (S1, S2, S3, S4, S5, S6) felt that private tutoring reduced the depth of their knowledge. They compared private tutoring with “feeding with a spoon.” Tutors fed them the lessons with a spoon and they swallowed those. One student remarked:

As the topic is not taught in detail in private tutoring, I face difficulty… I cannot sharpen my knowledge as I study in a private tutoring program using a short-cut method. I can merely fulfill my purpose with what I have learned in private tutoring (S3).

The country’s education policy is focused on the necessity of nurturing students’ ability to think critically. However, private tutors’ emphasis is on rote learning and examination skills rather than on developing their critical thinking skills (Islam et al., 2021, p. 746). Moreover, private tutors supplied hand-notes and suggestions. Because of getting everything ready-made, students become dependent on tutors by losing their own creativity. Their tendency to work hard was being ruined. Two student interviewees reported:

We, the students, are not doing anything on our own… Private tutoring is diminishing our creativity. It is destroying our mentality of hard work (S6).

The class teacher did not solve all the problems. But in private tutoring, whenever I cannot understand something, I show it to the private tutor and he solves it. So, I do not have to solve it myself. We are losing our creativity and we are not practicing on our own (S4).

Similarly, all parent interviewees (P1, P2, P3, P4, P5, P6) opined that private tutoring helped their children improve their examination scores, but it actually decreased their talent. Their children did not try to solve the problems by themselves first. Rather, they were becoming dependent on others:

Students are becoming dependent on teachers… Each student has to go to a different teacher for each subject. My daughter is now taking tutoring from three different teachers. This is a matter of anxiety (P4).

Two parents (P1, P6) stated that students who missed the formal classes for private tutoring had some shortcomings. They noticed such shortcomings in their children’s studies too. Four teacher interviewees (T1, T2, T4, T5) perceived that they taught the students how to get better results in a shortcut way that suppressed their critical thinking skills. Two teacher interviewees (T3, T6) acknowledged the negative consequences of rote learning, but strategically claimed that it was unethical for them to suppress students’ merit. They covered all the contents of textbooks in private tutoring. They suggested that if private tutors provided their students with huge tasks, let them solve problems, and kept them engaged, then their creativity would not be diminished and they would not be dependent on others.

## Discussion

This study explored the academic effects of private tutoring in a Bangladeshi higher secondary educational context. Private tutoring has been documented as an essential input to survive in formal schooling (Akter et al., 2021, p. 340; Shonchoy and Rabbani, 2015, p. 7), as it is predicted to lead to better learning outcomes (Nath, 2012, p. 51). According to Transparency International Bangladesh, 44.4 percent of higher secondary students experienced the benefits of having private tutors (TIB, 2008, p. 22). The current study reported private tutoring as a supportive tool for higher secondary students, which eliminated their learning deficiencies and boosted their academic credentials. These findings are consistent with previous studies that found a positive correlation between students’ tutoring participation and their academic achievement (e.g., Mischo and Haag, 2002, p. 263; Dang, 2007, p. 684; Nath, 2008, p. 68; Ha and Park, 2017, p. 65; Shihab and Sultana, 2017, p. 9; Kuan, 2018, p. 391; Quadir, 2021, p. 215; Herath, 2022).

Academic achievement is one of the key goals for students. They must understand the subject matter to attain a better score in the examination. Mainstream schooling does not adequately stimulate their learning potential (Shekh, 2005, pp. 13–15). By contrast, private tutoring helps students overcome weaknesses in specific subjects, allowing them to perform well in examinations (Nath, 2007, p. 15). Furthermore, first-generation students in Bangladesh are rarely assisted with their studies beyond the classrooms (Xu et al., 2019, p. 34). Thus, private tutoring is essential for students who are struggling academically. In light of Vygotsky’s perspective, private tutors deliver personalized and individualized instruction. Students internalize subject-specific knowledge and skills through scaffolding by private tutors who are more knowledgeable or expert (Azmat et al., 2021, p. 277; Akani, 2015, p. 75; Hitchcock et al., 2004, p. 90). When proper scaffolding is provided, the learning becomes easier, students can solve their difficulties that they could not solve on their own (Bransford et al., 2000), and thus their academic achievement improves (Mohammed, 2019, p. 43).

The study also found private tutoring beneficial for helping higher secondary students succeed in the university entrance examination. Bangladeshi universities, especially public universities, have an extremely competitive enrollment process. Only academically most qualified students can be admitted into elite universities (Rahman et al., 2019a, p. 27; Alam and Zhu, 2022, p. 27). The HSC examination scores, in fact, decide the fates of students. A high score is required for getting a chance to study at a reputed university. In 2015, for example, only 6 percent of HSC examinees acquired GPA-5. In contrast, 66 percent of public university students were GPA-5 achievers on the HSC examination (World Bank, 2019, p. 27). As scaffolding through private tutoring is intended “to facilitate students’ upward academic mobility” (Kumar and Chowdhury, 2021, p. 245), their participation in it considerably impacts on their achievement in university entrance tests (Natasha et al., 2021, p. 320). Hajar and Abenova (2021, p. 124) found that 72 percent of Kazakhstani students used private tutoring as an enrichment strategy to enhance their scores on high-stakes examinations and thereby gain admission to university.

The findings further showed that private tutoring induced long-term deficiencies by encouraging rote learning. It dulled students’ critical thinking and made them dependent on others. These findings align with other studies, which revealed that private tutors focused more on drill learning than on critical thinking skills (Yung, 2020, p. 2; Azmat et al., 2021, p. 277). Bangladeshi students grow up in a credentialist society, where they value examination preparation for their learning (Sultana, 2019, pp. 130–135). They heavily rely on private tutoring to drill them with test-taking strategies. While scaffolding, private tutors only instructed on examination items or topics. This shortcut leads to more rote learning and less critical thinking (Fabee, 2019, p. 9). Thus, the students achieve high examination scores rather than improve their critical thinking skills.

Theoretically, scaffolding is useful for supporting students’ critical thinking as it allows them to become self-dependent learners (McCosker and Diezmann, 2009, p. 27). Many Bangladeshi parents, however, felt that their children had little prospect of passing an examination or getting better grades without private tutoring (Schuler, 2007, p. 190). They have largely supported the scaffolding instruction, although it comes with a financial burden for them (Mahmud, 2021, p. 47). While the world is moving to education sustainability and applicability, private tutoring in many contexts, including Bangladesh, still focuses on paper work and examination success rather than touching other critical skills necessary for students’ and community achievement. Therefore, scaffolding through private tutoring is often seen as unsuccessful or ineffective to support students’ critical thinking skills.

## Conclusion

Based on the findings of the present study, it can be concluded that private tutoring is an effective strategy to improve students’ academic outcomes, but it does not contribute to developing students’ critical thinking skills. However, an effective regulatory mechanism can be established to oversee the quality of private tutoring. More importantly, policymakers are urged to formulate realistic policies that strike a balance between the benefits of private tutoring and the necessity to safeguard students’ critical thinking abilities. Additionally, the effectiveness of classroom instruction can be strengthened as it can practically reduce the need for private tutoring. Parents are advised to consider the overall learning outcomes while adopting scaffolding instruction for their children.

To the best of the authors’ knowledge, this is the first study that sought to understand the academic effects of private tutoring from a Vygotskian philosophy of learning. Furthermore, the findings made a substantial contribution to the shadow education literature in Bangladesh, where there was little evidence on this issue and the effectiveness of private tutoring was being hotly debated. Policymakers, educational practitioners, and parents can consider and address the insights gained from this study while responding to private tutoring as an emerging practice.

The present study has some limitations. It was carried out in two research sites, only targeted on higher secondary students, and recruited a notably small sample size that reduced the depth and breadth of the findings. A large scale investigation is needed in multiple research sites, including urban cities, rural places, and other academic levels (e.g., primary and secondary). Most notably, this study investigated the academic effects of private tutoring based on students’ self-description. Thus, the current findings need to be supported by further studies.

## Data Availability Statement

The raw data supporting the conclusions of this article will be made available by the authors, without undue reservation.

## Ethics Statement

The studies involving human participants were reviewed and approved by Beijing Normal University. Written informed consent to participate in this study was provided by the participants’ legal guardian/next of kin.

## Author Contributions

MA and ZZ: conceptualization, methodology, formal analysis, and review and editing. MA: investigation and original draft preparation. ZZ: supervision and funding acquisition. All authors contributed to the article and approved the submitted version.

## Funding

This work was supported by the curriculum reform based on project-based learning (BNU00306-240200004).

## Conflict of Interest

The authors declare that the research was conducted in the absence of any commercial or financial relationships that could be construed as a potential conflict of interest.

## Publisher’s Note

All claims expressed in this article are solely those of the authors and do not necessarily represent those of their affiliated organizations, or those of the publisher, the editors and the reviewers. Any product that may be evaluated in this article, or claim that may be made by its manufacturer, is not guaranteed or endorsed by the publisher.
